# Changes in erythrocyte ATPase activity under different pathological conditions

**DOI:** 10.4314/ahs.v17i4.31

**Published:** 2017-12

**Authors:** Ali A Kherd, Nawal Helmi, Khadijah Saeed Balamash, Taha A Kumosani, Shareefa A AL-Ghamdi, M Qari, Etimad A Huwait, Soonham S Yaghmoor, Alaama Nabil, Maryam A AL-Ghamdi, Said S Moselhy

**Affiliations:** 1 Biochemistry Department, Faculty of Science, King Abdulaziz University, Jeddah, Kingdom of Saudi Arabia; 2 Experimental Biochemistry Unit, King Fahd Medical Research center KFMRC, Jeddah, Kingdom of Saudi Arabia; 3 Production of natural products for Industrial health Research group, King Abdulaziz University, Jeddah, Kingdom of Saudi Arabia; 4 Hematology department, Faculty of Medical Science, King Abdulaziz University, Jeddah, Kingdom of Saudi Arabia; 5 King Abdulaziz University hospital, Jeddah, Kingdom of Saudi Arabia; 6 Bioactive Natural Products Research Group, King Abdulaziz University, Jeddah, Kingdom of Saudi Arabia; 7 Biochemistry Department, Faculty of science, Ain Shams University, Cairo, Egypt

**Keywords:** Na^+^-K^+^ATPase, red blood cell, pregnancy, smoking, diabetes, kidney diseases

## Abstract

**Background:**

Studies have shown that Na^+^-K^+^ ATPase activity was altered in disrupted red blood cell membranes and this enzyme is believed to be the site of active transport of Na^+^ and K^+^ in intact red blood cells. The enzyme is often referred to as Na^+^-K^+^ pump because it pumps Na^+^ out and K^+^ into the cell against gradients with the concomitant hydrolysis of intracellular ATP.

**Objective:**

The aim of this study was to find out the possibility of using Na^+^-K^+^-ATPase activity as a biomarker for the diagnosis of individuals with different physiological conditions.

**Materials and methods:**

The activity of Na^+^-K^+^ ATPase was determined in blood samples collected from different pathological and physiological conditions such as pregnancy, smoking, diabetes and renal dysfunction compared with healthy subjects matched for age and sex.

**Results:**

The Na^+^-K^+^ ATPase activity in pregnancy (0.094 ± 0.0051 µM Pi/min. mg protein), smoking (0.064 ± 0.0011 µM), diabetes (0.047 µM 0.002 µM) and kidney disease (0.069 ± 0.0014 µM) was higher compared to the measurements in healthy individuals (0.0081 ± 0.0031 µM).

**Conclusion:**

Na^+^-K^+^ATPase specific activity is a biomarker for the diagnosis of individuals with different physiological diseases.

## Introduction

Studies have shown that Na^+^-K^+^-ATPase activity was altered in disrupted red blood cell membranes^1^ and this enzyme can be the site of active transport of Na^+^ and K^+^ in intact red cells^2^. The enzyme is often referred to as Na^+^-K^+^ pump because it pumps Na^+^ out and K^+^ into the cell against gradients with the concomitant hydrolysis of intracellular ATP^3^. There are three types of ATPase: P-type ATPases, V-type ATPases and ABC transporters for ATP binding cassette. The distinguishing functional characteristic of P-type ATPases is that the hydrolysis of ATP that occurs during the catalytic cycle results in the phosphorylation of the transport protein itself. This phosphorylation results in a change in the conformation of the protein that, in turn, plays a part in the translocation of one or more substrate molecules across the membrane^4^.

Erythrocyte Na^+^, K^+^-ATPase, and Ca-^2+^, Mg-^2+^-ATPase activity in young individuals were markedly lower than that in the adults. Intra-erythrocyte free Ca-^2+^ concentration in young individuals was markedly higher than that in adults^3^. The two ATPases activity in the elderly group over 60 years old were lower than that in young individuals, intra-erythrocytic free Ca-^2+^ concentration in the elderly group was higher than that in young individuals. In all age groups, the three parameters mentioned above in young individuals showed most remarkable change with aging and that changes in young females were more remarkable than that in young males^5^.

Placenta showed an increase of Na^+^, K^+^ -ATPase on day 21 of gestation in comparison to day 12^6^. Erythrocyte Na^+^, K^+^-ATPase activity was normal in pregnant women, suggesting that the peripheral metabolic status during pregnancy was normal. Increases in both the number and function of the pump may be influenced by factors other than thyroid function^7^.

The red-blood cell membrane possesses an ATP-dependent transport system by which the hydrolysis of one molecule of ATP results in the linked movement of three Na^+^ ions out of the cell and two K^+^ ions into the cell. This active transport process is dependent upon the appropriate concentrations of Na^+^ and K^+^ inside and outside of the cell membrane, and is specifically inhibited by low concentrations of cardiac glycosides^8^.

The Na^+^ transport system represents a complex protein-lipid moiety appropriately orientated within the membrane. The number of Na^+^ and K^+^ transport sites per red cell has been calculated from kinetic data and from the binding of tritium-labeled ouabain to be approximately 200 per cell 9–11.

The rationale for this study was the possible utility of erythrocyte ATPase as a biomarker of physiological changes in human bodies. This will help in early diagnosis of health status.

The aim of this study was to find out the possibility of using Na^+^-K^+^ATPase specific activity as a biomarker for the diagnosis of individuals with different physiological and pathological conditions.

## Subjects and methods

Sucrose, magnesium chloride hexahydrate, ascorbic acid, sodium chloride, potassium chloride, ammonium molybdate, ethylenediaminetatra-acetic acid (EDTA), sodium hydroxide, sodium dodecyl sulphate (SDS), sulfuric acid , hydrochloric acid and Tris tris [hydroxymethyl] aminoethane were purchased from BDH limited Poole, England. Adenosine-5′- triphosphate disodum salt and ouabain were obtained from Sigma Chemical Company St. Louis, MO, USA.

### Subjects

Human subjects included in this study were selected from four different hospitals in Jeddah area of Saudi Arabia, King Abdulaziz University Hospital, King Fahd Hospital, Al Thaqer Hospital and Maternity Hospital.

Total of 149 subjects were included in this study and divided into two main groups. The first group consisted of 63 normal healthy individuals of the age 23–50 years and this group was used as a control. This control group consisted of 34 males and 27 females. The second group (n=86) consisted of subjects with physiological / conditions which include pregnancy (n=21), smoking (n=30) and pathological conditions such as diabetes (n=20) and kidney disease (n=15). Detailed history, mental, physical status and clinical examination showed that the control group consisted of completely healthy individuals and had not been subject to any therapeutic drugs during the past 4 months of blood sampling. Patients with different physiological changes were confirmed by clinical and biochemical examinations. The subjects were informed of their consent prior to drawing blood samples.

A venous blood sample 5ml was collected in a heparin's tube from each subject and kept in an icebox. Plasma was separated by centrifugation at 4500 rpm at 4°C. Precipitated RBC's were washed by isotonic solution 0.9 % w/v NaCl, throughout; this step was repeated three times. Another wash with de-ionaized water was performed, and then stored at −20°C overnight. After thawing, the supernatant was separated by centrifugation at 8000 rpm for 30 min, and RBC was used for the Na^+^-K^+^ ATPase assay.

### Determination of the Na^+^, K^+^ -ATPase activity

The ATPase activity was quantified by measuring the release of Pi from ATP Serrano, 1978. A spectrophotometric method was adopted, the quantity of Pi in the assay was then determined spectrophotometrically against a standard curve derived from solution of known phosphate concentration^12^. One ml of RBC homogenate was added to ATPase buffer 50 mM Tris, 3 mM MgCl2. 6H2O, 10 mM KCl, 0.1 mM EDTA, 100 mM NaCl at pH 7.4 and ^+^/- Ouabine. Incubated for 5 min at 37oC in water bath then 2 mM ATP was added and incubated further for 10 min. Ammonium molybdate solution 2 ml was added to stop the reaction^23^. By adding 20 µl of ascorbic acid, the absorbance of color complex was measured at 750 nm^13^. The calorimetric method was applied for the measurement of total protein according to the method of Biuret^14^.

## Statistical analysis

The data were logged into personal computer and analyses of data were performed using SPSS statistical package version 22. T-test was used for comparing means. P value was considered to be statistically significant if it was < 0.005.

## Results

**Figure 1:**
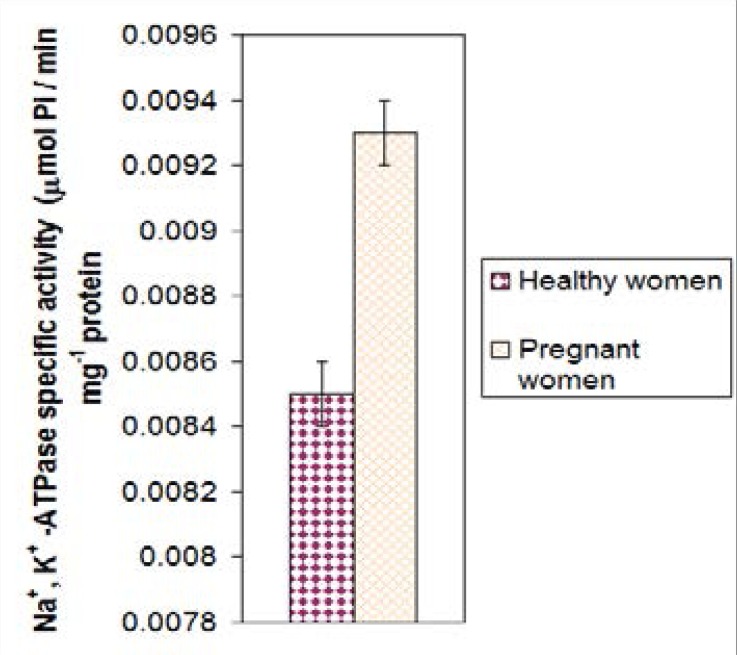
The mean values of Na^+^, K^+^ -ATPase specific activity of pregnant females compared to non-pregnant at same age (p<0.01).

In pregnancy, as shown in Figure 1, the mean activity of Na^+^, K^+^ -ATPase of healthy females (n= 21) was compared with pregnant women of the same age group. The mean of enzyme activity for pregnant women 0.0094 ± 0.001 nmol Pi/min. mg protein was higher than in healthy women 0.0086 ± 0.001 nmol Pi/min. mg protein and there was a highly significant difference between these two groups (P= 0.007).

**Figure 2:**
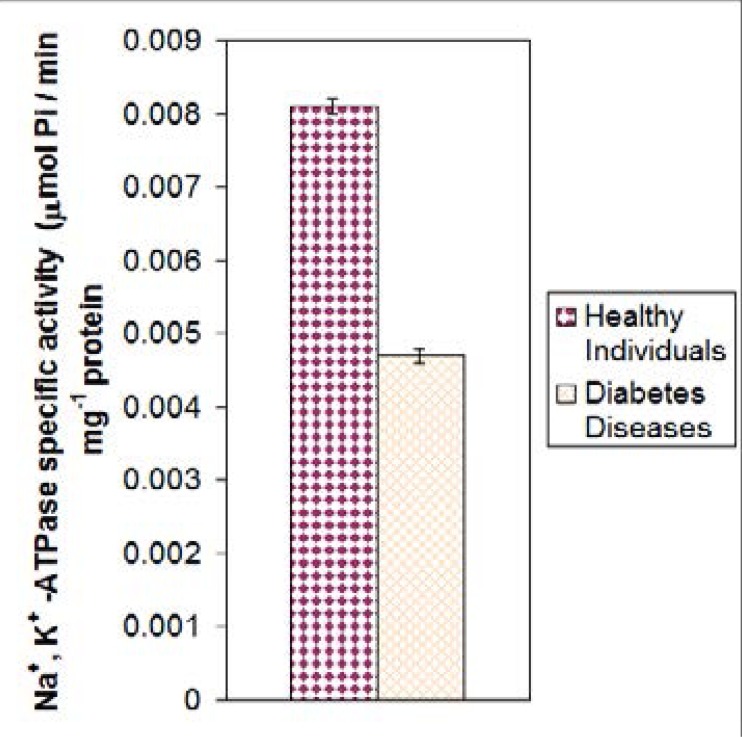
The mean values of Na^+^, K^+^ -ATPase specific activity of smokers compared to no smokers (p<0.001).

In smokers, as shown in Figure 2, the activity of Na^+^, K^+^ -ATPase for non-smokers individuals at ages 23–50 years was compared to smokers of the same age group. The enzyme activity of healthy, non-smokers 0.0081 ± 0.001 nmol Pi/min. mg protein was higher compared to smokers 0.0064 ± 0.001 nmol Pi/min. mg protein. There was highly significant difference between the two groups (P= 0.000).

**Figure 3:**
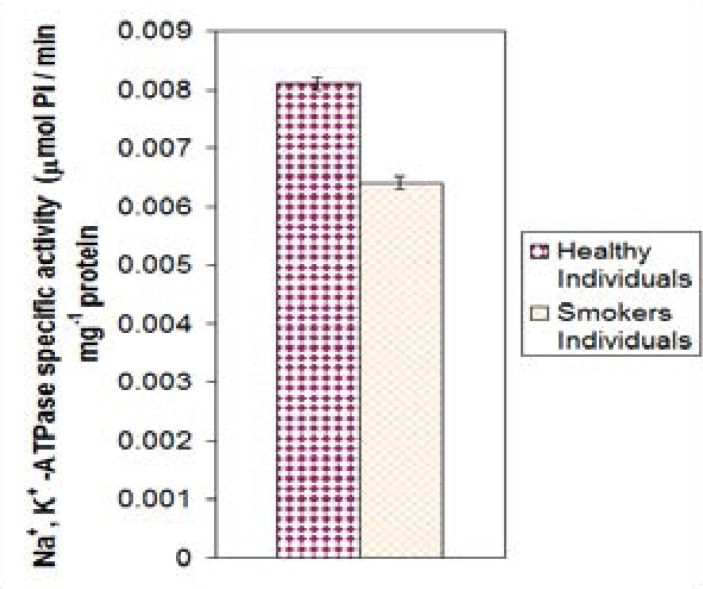
The mean values of Na^+^, K^+^ -ATPase specific activity of diabetes patient compared with normal at same age (p<0.01).

**Figure 4:**
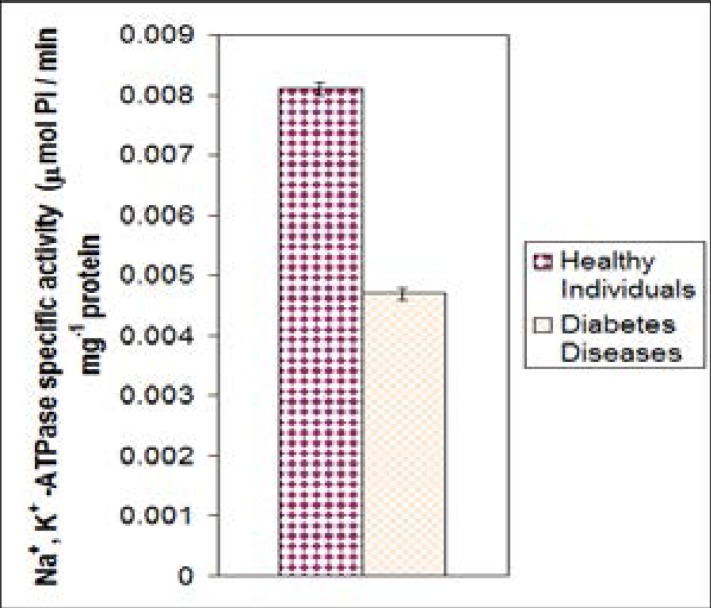
The mean values of Na^+^, K^+^ -ATPase specific activity of healthy males and females at age 23–50 years compared to kidney disease (p<0.05).

In diabetics, as shown in Figure 3 the enzyme activity of the patients was (n= 20) compared to healthy individuals (males and females) at 23–50 age group. The mean activity of Na^+^, K^+^ -ATPase for healthy group was 0.0081±0.001 nmol Pi/min. mg protein, which was higher than in diabetes patients (0.0047 ± 0.002 nmol Pi/min. mg protein) and the difference was highly significant P= 0.000. In kidney disease patients, as shown Figure 4 the enzyme activity of the n= 15 was compared with healthy individuals (males and females) at 23–50 age group. The mean activity of Na+, K+ -ATPase for healthy individuals was 0.0081 ± 0.001 nmol Pi/min. mg protein, which higher compared to kidney diseases patients 0.0069 ± 0.001 nmol Pi/min. mg protein and the difference was highly significant (P= 0.000).

No significant difference in ATPase activity between males and females.

## Discussion

The Na^+^/K^+^-ATPase is a membrane enzyme which is involved in the regulation of membrane potential, cell permeability of Na^+^, K^+^ and Ca^2+^ and excitatory neurotransmitters. It is also important in cell cycle and differentiation. The balance of Na^+^ and K^+^ between the intracellular and extracellular is the main requirement for cellular homeostasis and for different functions^3^. The rationale of this study was the possibility of utilization of Na^+^/K^+^-ATPase as a biomarker for blood diseases.

Previous reports stated that, there is an elevation of Na^+^, K^+^-ATPase activity in the primary anemia patients. This elevation may be a compensating mechanism for adaptation of the patients with low oxygen and its physiological role in the cell^14^.

It is assumed that any change in the micro-environmentof blood will have an effect on Na^+^, K^+^ -ATPase activity in the red cell membranes. To verify this assumption, the activity of Na^+^, K^+^ -ATPase from healthy individuals with different genders but same age group control were compared to those with different physiological conditions. Physiological changes in blood environment are related most of the time with alteration in enzyme specific activity which might be due to increased / decreased concentration of ions, biochemical materials or changes in membrane components and/or structures that is related to that changes.

The result obtained from this study indicated that the mean specific activity of Na^+^, K^+^ -ATPase of pregnant women is higher than the healthy women with highly statistically significant difference. This agrees well with published results^25^. This might be due to enzyme relative contribution either to metabolite transport process or/and to the thermogenesis during pregnancy. However, the increases in both the number and function of the pump may be influenced by factors other than thyroid function^26^. The mean value of specific activity of Na^+^, K^+^-ATPase for healthy individuals are higher compared to smokers of the same ages group and gender. This is in agreement with result obtained by Tulenko et al^25^, where they reported that the Na^+^, K^+^ -ATPase activity was decreased in smoker subjects compared with nonsmokers. The suppression in enzyme activity was related to increase in Na^+^ level inside cell. It was suggested that, chronic cigarette smoking causes changes in phospholipid bilayer in cell membrane of blood wall^26^.

The result obtained here indicated that the mean specific activity of Na^+^, K^+^ -ATPase of healthy individuals are higher than individuals with diabetes of the same age group and gender, which might be due to the insulin level. If insulin level is low, the rate of glycolysis in red cell become low and leads consequently to decrease the level of ATP so there would be a decreased in the enzyme activity^27^. In vitro insulin treatment of diabetic red cells was found to inhibit the further increase in its activity, but it failed to restore the activity to the normal level^26^.

The mean value of specific activity of Na^+^, K^+^ -ATPase for healthy individuals is higher compared to the kidney disease patients at the same age and gender, and the difference is highly significant. This might due to alteration in the ions in blood environment in kidney disease patients, which might lead to a decrease in enzyme specific activity^28^.
